# A Rare but Debilitating Disorder: A Case Report On Olfactory Reference Syndrome

**DOI:** 10.1192/j.eurpsy.2026.11802

**Published:** 2026-07-31

**Authors:** S. M. Alshahrani

**Affiliations:** Neurosciences, King Abdullah bin Abdulaziz University Hospital, Princess Nourah bint Abdulrahman University, Riyadh, Saudi Arabia

## Abstract

**Introduction:**

Olfactory reference syndrome (ORS) is persistent and false belief that one emits a foul, unpleasant, or socially offensive body odor lead to anxiety, depression, and significant functional impairment. Diagnosis remains challenging as its overlap with multiple psychiatric conditions. Despite DSM-5 categorized it as a part of “other specified obsessive-compulsive and related disorders,” its classification remains debated.

**Objectives:**

We aim to discus Olfactory reference syndrome (ORS) and its place in the DSM-5/ICD-11 diagnostic systems.

**Methods:**

We present this case study of a 45-year-old male with persistent foul mouth odor, accompanied by significant anxiety and repetitive hygiene behaviors that impacted his social and occupational functioning. Upon admission, physical and neurological examinations are normal. Mental status examination with Yale-Brown Obsessive-Compulsive Scale reflecting severe obsessive-compulsive symptoms. Patient was started on Cognitive Behavioral Therapy (CBT), with multiple interruptions and restarts of various psychiatric medications. Currently, He is on Aripiprazole 5 mg/day, Fluoxetine 20 mg/day, and a gradual tapering of Olanzapine with CBT.

**Results:**

**Table 1.** Table 1. Long description.

Proposed calcification for olfactory reference syndrome	
***Primary olfactory reference syndrome***	**When the condition does not have significant cause nor comorbid condition or substance use; it can be called primary olfactory reference syndrome.**
***Comorbid olfactory reference syndrome***	***When the condition has significant cause or comorbid psychiatric or medical condition or substance use; it can be called comorbid olfactory reference syndrome. Comorbid psychiatric conditions include obsessive compulsive disorder, depression, and anxiety disorders. Other medical conditions include neurological disorders and substance abuse.***

**Conclusions:**

This case study supports the perspective that olfactory reference syndrome is related to other specified obsessive-compulsive and related disorders classification. Future research should seek to delineate Olfactory reference syndrome (ORS) placewithin psychiatric classification systems.

**Disclosure of Interest:**

None Declared

## Long descriptions

### Table 1. Long description

The table is titled Proposed calcification for olfactory reference syndrome. It consists of two rows.

* Row 1: The left column identifies Primary olfactory reference syndrome. The right column defines it as a condition that does not have a significant cause, comorbid condition, or substance use.

* Row 2: The left column identifies Comorbid olfactory reference syndrome. The right column defines it as a condition that has a significant cause or comorbid psychiatric or medical condition or substance use. It specifies that comorbid psychiatric conditions include obsessive compulsive disorder, depression, and anxiety disorders, while other medical conditions include neurological disorders and substance abuse.

Navigate back to Table 1..

